# Decoding survival in MASLD: the dominant role of metabolic factors

**DOI:** 10.1186/s13098-025-01802-9

**Published:** 2025-06-18

**Authors:** Zhiqiang Jin, Cheng Zeng, Yang Yang, Shan Zhong, Zhi Zhou

**Affiliations:** 1 Department of Infectious Diseases, Key Laboratory of Molecular Biology for Infectious Diseases (Ministry of Education), Institute for Viral Hepatitis, The Second Affiliated Hospital, Chongqing Medical University, No 288, Tianwen Avenue, Chayuan, Nan’an District, Chongqing, 401336 China; 2 Chongqing Traditional Chinese Medicine Hospital, Chongqing, 400021 China

**Keywords:** MASLD, Metabolic factors, Prognosis, Machine learning algorithms

## Abstract

**Background:**

Metabolic factors are considered to influence disease progression in patients with Metabolic Dysfunction-Associated Steatotic Liver Disease (MASLD), but the impact of individual metabolic factors on the survival rate of patients with MASLD is still unclear.

**Aims:**

This article aims to reveal how metabolic components affect the survival of patients with this disease.

**Methods:**

A total of 3,086 participants with MASLD based on the diagnostic criteria established at the Delphi conference from NHANES III were included in this analysis. COX regression model (C-index = 0.64) was used to analyze the all-cause and attributable mortality of different number of metabolic factors. Elastic Network Regression model (C-index = 0.69), Accelerated Failure Time model and Randomized Survival Forest model (C-index = 0.63) based on machine learning were used to analyze the weight of each metabolic factor, and a Metabolism-related survival risk score formula was established and verified.

**Results:**

This study found that not only the number of metabolic factors had different effects on all-cause survival in MASLD patients, but also the degree of impact of different metabolic factors on survival was quite different, among which poor glycemic control was the most important influencing factor.

**Conclusion:**

This study highlights the clinical value of relevant metabolic factors in predicting survival in the MASLD patient population. Related metabolic factors can be used as surrogate biomarkers for the follow-up of MASLD patients.

**Supplementary Information:**

The online version contains supplementary material available at 10.1186/s13098-025-01802-9.

## Introduction

Metabolic dysfunction-associated steatotic liver disease (MASLD), has become a significant global health concern, affecting approximately 38% of adults worldwide [1, 2]. Previous study demonstrated that more than 30% of patients can progress to fibrosis and ultimately cirrhosis [3, 4], which significantly increases the risk of liver-specific mortality [5–7].

The nomenclature MASLD reflects the central role of metabolic dysfunction in disease pathogenesis. An increasing number of studies have shown that metabolic factors are responsible for the occurrence and development of this disease [8]. From the perspective of metabolic regulatory mechanisms in vivo, some metabolism-associated genes were found to participate in the progression of hepatic de novo lipogenesis and liver fat accumulation [9]. For example, the GPAM gene, which plays a role in metabolism, initiates triglyceride synthesis. A missense variant of this gene has been linked to fibrosis progression in MASLD patients [10], which may affect survival rates. The same idea has been demonstrated in clinical studies. Multiple large-scale cohort studies have demonstrated that metabolic factors affect fibrosis progression in MASLD patients [11, 12], which in turn affects their survival.

Recently, international experts, through a Delphi consensus process, suggested changing the current nomenclature as MASLD, thereby improving the assessment of metabolic factors in MASLD [13]. However, while current research indicates a potential link between metabolic traits and the advancement of MASLD, uncertainty persists regarding the correlation between metabolic traits and prognosis of MASLD. Thus, it is essential to investigate how metabolic traits impact the prognosis of MASLD patients and to address metabolic risk factors and improve risk stratification [14, 15].

This study presents a novel exploration into how metabolic profiles influence survival outcomes in patients with MASLD. By analyzing a cohort of 3,086 patients, the research uncovered that the prognostic outcomes vary significantly based on the number and combinations of metabolic factors present, despite the diagnostic flexibility in MASLD which allows for diagnosis based on a single metabolic factor. Hyperglycemia emerged as the most critical risk factor, followed by hypertension, with other factors showing comparatively lesser impact. Innovatively, a machine learning algorithm was utilized to develop a scoring formula that enables a more scientific and precise stratification of metabolic risk among these patients, enhancing the predictive accuracy of treatment outcomes. This approach marks a significant advancement in the personalized management and treatment of MASLD.

## Materials and methods

### Population included

**Fig. 1:**
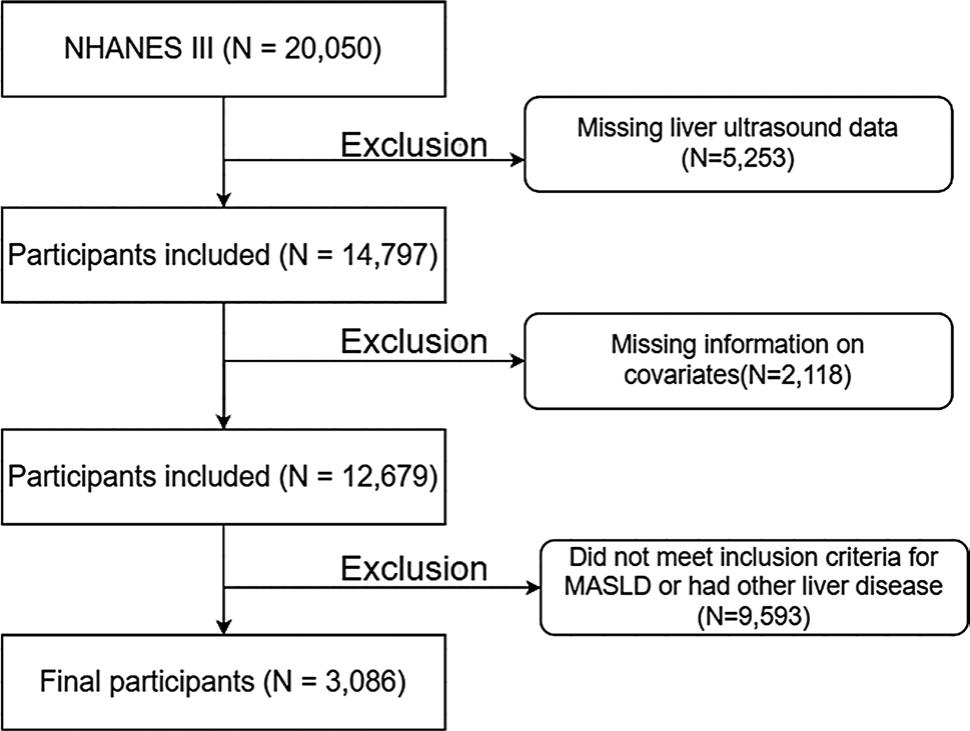
Flow chart of data cleaning and inclusion of participants

In this study, the MASLD population was identified retrospectively using the NHANES. The population health data and analysis methods of NHANES III can be accessed publicly on the website. Survival data for the screened MASLD population are linked to the public-use mortality file. The research protocol was approved by the Research Ethics Review Board of the Centers for Disease Control and Prevention, and written informed consent was obtained from all participants. In this study, participants in NHANES III cohort (*N* = 20,050) underwent a stepwise exclusion process as illustrated in Fig. 1. Firstly, 5,253 individuals were excluded because of the incomplete liver ultrasound data. Subsequently, 2,118 participants were excluded due to missing covariates. Finally, 9,593 participants were excluded as they did not meet the predefined inclusion criteria for MASLD or had evidence of other liver diseases. This resulted in a final analytic sample of 3,086 participants.

### Covariates

Covariates assessed for inclusion in this analysis included age, gender, race, education level, poverty-to-income ratio (PIR), smoking, alcohol use, BMI, waist circumference, blood pressure measurement, and test results such as fasting glucose, glycosylated haemoglobin, triglycerides, HDL-cholesterol, and alanine aminotransferase (ALT).

### Diagnosis of MASLD

In NHANES III participants, hepatic steatosis was identified using a Hepatic Steatosis ultrasound examination. In alignment with previous research [16, 17], This study considered participants to have hepatic steatosis if they exhibited mild, moderate or severe hepatic steatosis.

After hepatic steatosis was identified by ultrasonography, we screen out patients with MASLD according to a new fatty liver disease nomenclature according to a multisociety Delphi consensus statement, It was decided that patients with steatosis and any cardiometabolic criteria would be diagnosed with MASLD [13], which was agreed that patients with steatosis and any one of the cardiometabolic criteria including:1)BMI ≥ 25 kg/m²[23 Asia] OR WC > 94 cm (M) 80 cm (F); 2)Fasting serum glucose ≥ 5.6 mmol/L [100 mg/dl] OR 2-hour post-load glucose levels ≥ 7.8 mmol/L [≥ 140 mg/dl] OR HbA1c ≥ 5.7% [39 mmol/L] OR type 2 diabetes OR treatment for type 2 diabetes; 3)Blood pressure ≥ 130/85 mmHg OR specific antihypertensive drug treatment; 4)Plasma triglycerides ≥ 1.70 mmol/L [150 mg/dl] OR lipid lowering treatment; 5)Plasma HDL-cholesterol ≤ 1.0 mmol/L [40 mg/dl] (M) and ≤ 1.3 mmol/L [50 mg/dl] (F) OR lipid lowering treatment would be considered to have MASLD.

### Status of survival

In order to determine survival status, the Public-Use Linked Mortality File was used, which is linked to the National Death Index (NDI) data via a probability matching algorithm and NHANES data is linked to the NDI. Deaths occurring before 1999 were classified according to ICD-9 codes, and deaths occurring after 1999 were classified according to ICD-10 codes.

### Machine model selection

In this study, elastic network regression, accelerated time to failure (AFT) model and random forest model were selected to assess the relationship between independent variables and survival outcomes. Elastic network regression combined with L1 and L2 regularisation can effectively deal with multicollinearity and variable selection, and provide stable regression coefficient estimates. The AFT model directly models survival time and can explain the additive multiplicative effect of the independent variables on survival time, which is complementary to the COX model and avoids the one-sidedness of a single model. The Random Forest model, as an integrated learning approach, is able to deal with non-linear relationships and variable interactions, providing an independent assessment of significance. These models complement each other in validation to ensure the robustness and reliability of the results.

### Statistical analysis

In this paper, analysis of data is conducted using R (4.4.1) and relevant R packages (survival 3.7-0, glmnet 4.1-8 and other data processing packages, like the survey package: incorporating survey weights from NHANES III’s complex survey design). A Kruskal-Wallis rank sum test and chi-square test were used to assess differences between the two groups. Variables were presented as means ± SD and frequency/percentage, with statistical significance set at *P* < 0.05.

The Hazard ratio (HR) and 95% confidence intervals (CIs) between metabolic factors and mortality were estimated by Multivariable COX regression model. Three multivariable COX regression models were constructed, and metabolic factors were categorized into five groups based on cumulative numbers, with the group having only one metabolic trait serving as the reference.

A formula was constructed using Elastic Network model based on machine learning algorithms to calculate the weights of each metabolic factor. The magnitude of the weights was verified using Accelerated Failure Time model and Randomized Survival Forest model.

## Results

### Baseline data for participants categorized by metabolic factors

Of the 20,050 adults in NHANES III, after excluding null values of covariates and other relevant missing indicators, we included 3,086 patients in this article with MASLD according to the recommended diagnostic criteria. As Fig. 1 illustrates the specific screening process.

The baseline characteristics of participants with different metabolic factors are shown in Table 1. The number of metabolic factors in the screened patients are assessed and categorised them into groups M1, M2, M3, M4 and M5 on the basis of the number of cardiometabolic criteria for which they fulfilled the Delphi consensus statement(e.g. inclusion in Group M1 if only one metabolic criterion is met). As shown in Table 1, age, race, education level, smoking, and metabolism-related indicators such as abdominal obesity, body mass index (BMI), hypertension, diabetes, serum triglycerides, and HDL cholesterol were significantly different between the groups. Among the five groups, participants in the lower metabolic factors group were significantly younger and more likely to have higher education. They were also less likely to have a history of smoking, and they had lower prevalence of hypertension and diabetes, few abdominal obesity and BMI, and tended to have lower triglyceride (TG) levels and higher HDL-cholesterol levels.

**Table 1:** Baseline characteristics of study participants according to different metabolic factors, NHANES III (1988–1994)

Characteristic	M1 *N* = 418^1^	M2 *N* = 610^1^	M3 *N* = 827^1^	M4 *N* = 755^1^	M5 *N* = 476^1^	*p*-value^2^
**Age (years)**	38 (± 14)	45 (± 15)	49 (± 15)	54 (± 14)	57 (± 13)	< 0.001
**Sex**						0.002
Female	264 (63%)	361 (59%)	446 (54%)	396 (52%)	267 (56%)	
Male	154 (37%)	249 (41%)	381 (46%)	359 (48%)	209 (44%)	
**Race/ethnicity**						0.001
Mexican American	143 (34%)	219 (36%)	297 (36%)	310 (41%)	208 (44%)	
Non-Hispanic White	126 (30%)	226 (37%)	281 (34%)	272 (36%)	153 (32%)	
Non-Hispanic Black	15 (3.6%)	26 (4.3%)	34 (4.1%)	32 (4.2%)	12 (2.5%)	
Other races	134 (32%)	139 (23%)	215 (26%)	141 (19%)	103 (22%)	
**Poverty income ratio**						0.302
< 1.3	131 (31%)	192 (31%)	245 (30%)	217 (29%)	145 (30%)	
1.3–3.49	171 (41%)	233 (38%)	361 (44%)	312 (41%)	214 (45%)	
> 3.5	116 (28%)	185 (30%)	221 (27%)	225 (30%)	117 (25%)	
**Education level**						< 0.001
≤ High school	262 (63%)	460 (75%)	620 (75%)	583 (77%)	385 (81%)	
> High school	156 (37%)	150 (25%)	207 (25%)	172 (23%)	91 (19%)	
**Smoking exposure**	166 (40%)	262 (43%)	397 (48%)	396 (52%)	264 (55%)	< 0.001
**Ever drank alcohol**	92 (22%)	129 (21%)	164 (20%)	174 (23%)	106 (22%)	0.609
**BMI**,** kg/m2**						< 0.001
30	72 (17%)	201 (33%)	407 (49%)	405 (54%)	285 (60%)	
25–30	101 (24%)	246 (40%)	327 (40%)	308 (41%)	171 (36%)	
25	245 (59%)	163 (27%)	93 (11%)	42 (5.6%)	20 (4.2%)	
**abdominal obesity**	96 (23%)	257 (42%)	402 (49%)	413 (55%)	296 (62%)	< 0.001
**Hypertension**	74 (18%)	229 (38%)	490 (59%)	555 (74%)	470 (99%)	< 0.001
**Diabetes**	7 (1.7%)	36 (5.9%)	141 (17%)	245 (32%)	247 (52%)	< 0.001
**HDL-cholesterol**	55 (± 14)	52 (± 15)	46 (± 13)	42 (± 12)	37 (± 7)	< 0.001
**Triglycerides**	93 (± 38)	124 (± 70)	167 (± 98)	231 (± 139)	287 (± 144)	< 0.001
**ALT**,** U/L**	25 (± 86)	22 (± 51)	31 (± 93)	30 (± 79)	32 (± 90)	< 0.001

^1^Mean (±SD) or n (%) N:participants number ^2^Kruskal-Wallis rank sum test; Pearson's Chi-squared test

### The relationship between metabolic factors and mortality

#### COX regression analysis

Table 2 presents the associations between different numbers of metabolic factors and patient survival using three multivariable logistic regression models: Model 1, without any covariate adjustments; Model 2, adjusted for age, sex, race/ethnicity, poverty degree, education level, and smoking status; Model 3, adjusted for age, sex, race/ethnicity, poverty degree, education level, smoking status, and alanine aminotransferase (ALT). In Model 3, compared to the M1 reference group, we found that an increase in metabolic factors was associated with higher all-cause mortality in MASLD patients (HR 1.67, 95%CI 1.32–2.12 for M3; HR 2.02, 95%CI 1.59–2.55 for M4; and HR 2.26, 95%CI 1.77–2.88 for M5). However, there was no significant difference between the M2 group and the M1 group. When M2 was used as the reference, the all-cause mortality in the M3, M4, and M5 groups was higher. (HR 1.43, 95%CI 1.22–1.67 for M3; HR 1.72, 95%CI 1.48-2.00 for M4; and HR 1.93, 95%CI 1.63–2.27 for M5). Meanwhile, the test for trend was also statistically significant (P for trend < 0.001), which demonstrated that a progressive increase in risk correlating with more metabolic factors. However, as shown in Supplementary Table 1, the number of metabolic factors was not significantly associated with specific causes of death, such as cardiovascular and neoplasms, when attribution analyses were performed.

**Table 2:** Hazard ratios for different number of metabolic factors in patients with MASLD

	Model 1			Model 2			Model 3		
**Metabolic Group**	**HR** ^1^	**95% CI** ^1^	**p-value**	**HR** ^1^	**95% CI** ^1^	**p-value**	**HR** ^1^	**95% CI** ^1^	**p-value**
M1	Reference			Reference			Reference		
M2	1.74	1.35, 2.24	< 0.001	1.26	0.98, 1.63	0.074	1.26	0.98, 1.63	0.074
M3	2.58	2.04, 3.27	< 0.001	1.67	1.32, 2.12	< 0.001	1.67	1.32, 2.12	< 0.001
M4	3.71	2.94, 4.68	< 0.001	2.02	1.59, 2.56	< 0.001	2.02	1.59, 2.55	< 0.001
M5	5.01	3.95, 6.37	< 0.001	2.26	1.77, 2.89	< 0.001	2.26	1.77, 2.88	< 0.001
*P* for trend			< 0.001			< 0.001			< 0.001
M2	Reference			Reference			Reference		
M3	1.82	1.55, 2.12	< 0.001	1.43	1.22, 1.67	< 0.001	1.43	1.22, 1.67	< 0.001
M4	2.61	2.24, 3.03	< 0.001	1.72	1.48, 2.01	< 0.001	1.72	1.48, 2.00	< 0.001
M5	3.52	3.00, 4.14	< 0.001	1.93	1.64, 2.27	< 0.001	1.93	1.63, 2.27	< 0.001
*P* for trend			< 0.001			< 0.001			< 0.001

Adjust:

Model 1: Unadjusted

Model 2: Adjusted for age, sex, race/ethnicity, poverty degree, education level, smoking status;

Model 3: Adjusted for age, sex, race/ethnicity, poverty degree, education level, smoking status, and alanine aminotransferase (ALT);

#### Survival analysis

**Fig. 2:**
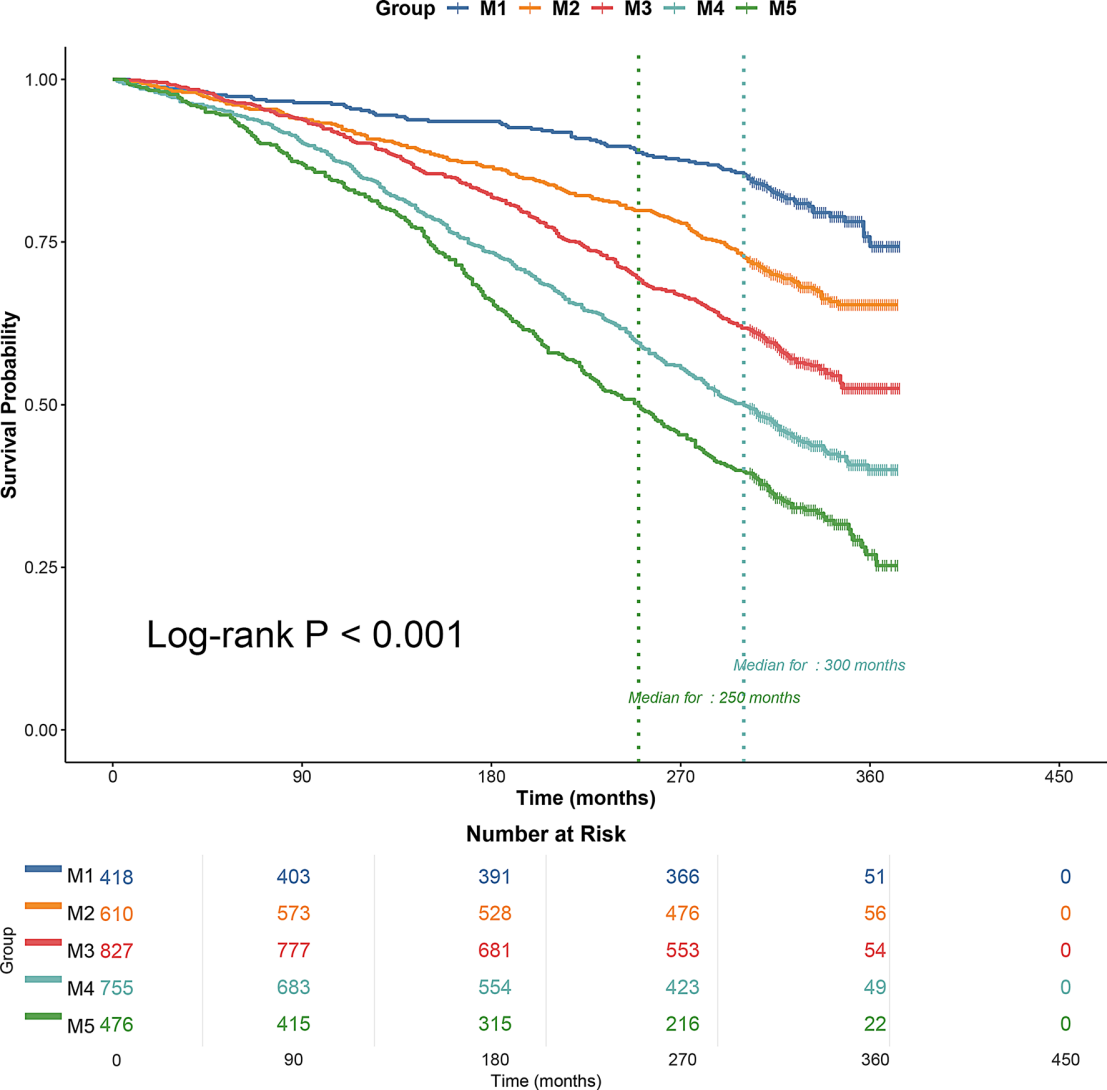
Kaplan-Meier survival curves grouped by the number of metabolic factors (from one to five). Patients were categorized into groups based on the number of metabolic factors they presented. Survival probabilities over time are shown for each group, illustrating the relationship between the number of metabolic factors and survival outcomes. Higher numbers of metabolic factors are associated with lower survival probabilities

Survival data were analyzed using the R package, and Kaplan-Meier survival curves were plotted to show survival rates for the M1-M5 group of participants. All-cause mortality decreased progressively as the number of metabolic factors decreased in Fig. 2.

In addition, the included MASLD participants were grouped by different metabolic factor types. As shown in Supplementary Fig. 1, patients with hypertension or diabetes mellitus were significantly more likely to die from all causes, whereas the other three metabolic factors were not significantly different. Further, as shown in Supplementary Fig. 2, participants were stratified by the number of different metabolic factors, and survival rates were analyzed for combinations of different metabolic factor types. It was observed that patients with comorbid hypertension and diabetes mellitus in the different stratified groups had significantly lower survival rates.

#### Subgroup analysis

**Fig. 3:**
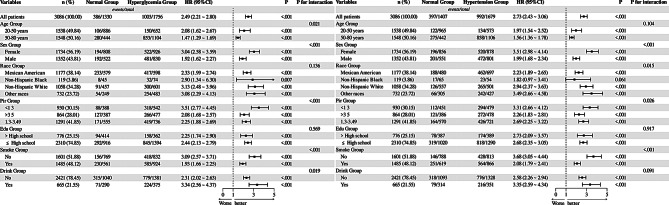
Subgroup analyses of hypertension (HBP) and hyperglycemia (GLU) in MASLD patients. Comparing all-cause mortality between affected and unaffected groups. Forest plots display HRs with 95% CIs (statistical significance if CI excludes 1). P values assess association strength; P for interaction tests subgroup effect differences. Elevated HRs indicate stronger metabolic factor-mortality links

Analyze the relationship between metabolic factors and the survival of patients with MASLD under conditions of different age stages, gender, ethnicity, poverty, educational status, and smoking or drinking status, we conducted subgroup analyses of the above factors. As shown in Fig. 3, when the exposure metabolic factor was hypertension, the relationship between metabolic factors and their survival was not significant in the non-Hispanic black population (HR = 1.82, 95%CI: 0.97–3.41, *P* = 0.061). In contrast, the relationship was significant for all other stratification factors (*P* < 0.01). In addition, for the survival rate of MASLD, an interaction was observed between hypertension and gender, race, poverty levels, and smoking. There were also interactions between hyperglycemia and age, gender, poverty levels, smoking and drinking (P for interaction < 0.05). Interestingly, nonsmokers with hypertension or hyperglycemia had a significantly higher risk of survival (HR = 3.68 and HR = 3.09, respectively), this paradoxical association may arise through three interrelated mechanisms: First, survivor bias could lead to selective attrition of smokers with severe comorbidities prior to follow-up. Second, smoking’s pleiotropic effects on inflammatory suppression (e.g., nicotine-mediated NF-κB inhibition) and catecholamine regulation might transiently mask metabolic dysfunction. Third, critical residual confounding may exist in three domains: (1) unmeasured behavioral gradients (e.g., differential healthcare utilization patterns and circadian disruption in smokers), (2) pharmacological effect modifiers (e.g., interaction between smoking-induced cytochrome P450 activation and oral hypoglycemic drugs), and (3) epigenetic aging acceleration that differentially modulates metabolic resilience. These unresolved complexities position our findings as a catalyst for three research frontiers: First, the paradox highlights the need for dynamic exposure-disease frameworks incorporating stochastic mortality competing risks. Second, it necessitates multi-omics investigations to disentangle smoking’s dual role as both toxin and biological response modifier. Finally, it underscores the imperative of developing exposure-specific comorbidity clocks that account for metabolic hysteresis effects. In addition, although the interaction between drinking and hypertension did not reach statistical significance (P for interaction = 0.091), stratified analyses showed a significantly higher risk of survival in hypertension patients with drinking (HR = 3.35, 95% CI: 2.59–4.34), while the interaction between drinking and hyperglycemia is statistical significance (P for interaction = 0.019) and stratified analyses also showed a significantly higher risk of survival in hyperglycemia patients with drinking (HR = 3.34, 95% CI: 2.56–4.37). In racial subgroup analyses, the association between hypertension and the risk of death among non-Hispanic black patients did not reach statistical significance (HR = 1.82, 95%CI: 0.97–3.41, *P* = 0.061). In contrast, Mexican Americans, non-Hispanic whites, and other races showed significant associations. This heterogeneity may reflect the fact that genetic predisposition (e.g., APOL1 variants among blacks), socioeconomic factors, or healthcare accessibility such as systemic barriers to healthcare access, low insurance coverage, and inadequate primary care may delay the diagnosis and management of metabolic disorders, resulting in confounders (e.g., uncontrolled comorbidities) that mask the true association. Future studies should explore whether culturally adapted interventions, such as community-based BP monitoring, can reduce disparities in MASLD outcomes.

In Supplementary Fig. 3, metabolic factors such as abdominal obesity, higher TG, and lower HDL-cholesterol levels appeared to be insignificantly associated with mortality in MASLD participants in the appeal subgroup.

### The weight of each metabolic factor was evaluated by machine learning algorithm model

#### Elastic net regression (ENR) model

A machine learning-based metabolic risk scoring system was developed using Elastic Net Regression to quantify the predictive contributions of metabolic factors on survival outcomes. This method combines variable selection with regularization, effectively balancing model accuracy and generalizability. The final risk score was derived from weighted combinations of key metabolic factors, with coefficients presented in Table 3.

**Table 3:** The weight of each metabolic factor was evaluated by different machine learning models model 1: elastic net regression (ENR) model

Variable	Units	Coefficients
Mean arterial pressure	mmHg	0.022144
Glucose	mmol/L	0.070084
Waistline	cm	0.008983
HDL-cholesterol	mg/dL	0.004648
Triglycerides	(mg/dL)	0.000583

Formula:

Met-score = β1 × Mean arterial pressure + β2 × Glucose + β3 × Waistline + β4 × HDL-cholesterol + β5 × Triglycerides

All metabolic factors were standardised to improve ensure that the contribution of all variables to the score was measured in terms of their significance rather than their magnitude or unit, avoiding the influence of data scale on the model. Key variables via coefficient shrinkage and constructs an interpretable linear formula

βi = Coefficients

**Model 2:** Accelerated Failure Time (AFT) Model

Variable	Units	Coefficients	Acceleration Factor (e^coefficient^)
Mean arterial pressure	mmHg	−0.012004	98.57%
Glucose	mmol/L	−0.400049	95.61%
Waistline	cm	−0.004223	99.41%
HDL-cholesterol	mg/dL	−0.000321	99.67%
Triglycerides	(mg/dL)	−0.004082	99.96%

Negative coefficients indicate that these factors are associated with a reduction in survival time. The absolute magnitude of the coefficients indicates the strength of the effect, and these values are converted to acceleration factors by means of indices

Acceleration Factor = e^Coefficients​^

**Model 3:** Randomised survival forest (RSF) model

Variable	Units	Importance	Comparative Importance
Mean arterial pressure	mmHg	0.113667	0.791
Glucose	mmol/L	0.143742	1.000
Waistline	cm	0.002718	0.019
HDL-cholesterol	mg/dL	−0.001273	−0.009
Triglycerides	(mg/dL)	0.021256	0.148

Comparative Importance = Importance / Importance_(Glucose)_, represents importance relative to diabetes weight

**Formula**:

$$\begin{gathered} Met - score{\text{ }} = {\text{ }}\beta 1{\text{ }} \times {\text{ }}Mean{\text{ }}arterial{\text{ }}pressure{\text{ }} \hfill \\+ {\text{ }}\beta 2{\text{ }} \times {\text{ }}Glucose + {\text{ }}\beta 3{\text{ }} \times {\text{ }}Waistline + {\text{ }}\beta 4{\text{ }} \hfill \\\times {\text{ }}HDL - cholesterol + {\text{ }}\beta 5{\text{ }} \times {\text{ }}Triglycerides \hfill \\ \end{gathered}$$

Model optimization involved ten-fold cross-validation to determine the regularization parameter λ (lambda = 0.005030), which achieved minimal prediction error without overfitting (Supplementary Fig. 4). Patients were subsequently stratified into four risk quartiles (Q1-Q4) based on their metabolic scores.

Ultimately, we calculated the metabolic risk score for this population based on the metabolic score formula in Table 3 and presented the survival curves based on the metabolic risk score quartiles in Supplementary Fig. 5. The Kaplan-Meier curves demonstrated progressively worse survival outcomes across ascending risk quartiles (log-rank test *P* < 0.001), with clear separation between adjacent quartiles. The sample was divided into four groups: Q1 (lowest risk), Q2, Q3, and Q4 (highest risk). These groupings revealed differences in survival probabilities at different risk levels, with the Q1 group having significantly higher survival probabilities than the Q4 group. There was a significant decrease in the number of high-risk individuals (Q4), compared to the low-risk group (Q1), further confirming the strong association between higher metabolic scores and lower survival probabilities and demonstrating that the metabolic score calculation formula we constructed is scientifically sound. This graded relationship visualized in Supplementary Fig. 5 was quantitatively corroborated by the hazard ratios in Supplementary Table 2 with detailed information on hazard ratios (HR) for each risk subgroup, as well as 95% confidence intervals (CI). Specifically, compared to the lowest risk group Q1, the hazard ratios were 1.65 (95% CI: 1.38, 1.98, *p* < 0.001) for group Q2, 2.47 (95% CI: 2.08, 2.93, *p* < 0.001) for group Q3, and as high as 3.65 (95% CI: 3.09, 4.31, *p* < 0.001) for the highest risk group Q4. In the adjusted model 3, the test for trend is still statistically significant (P for trend < 0.001).These data not only emphasize the importance of metabolic scoring in predicting survival probability but also support the potential clinical value of this scoring system.

#### Accelerated failure time (AFT) model

In order to reassess and validate the magnitude of the weight of each metabolic factor, an AFT model based on a machine learning algorithm was used to assess the effect of each metabolic factor on the logarithmic scale of survival time, and the correlation coefficients are shown in Table 3. In addition, the acceleration factors were obtained by applying an exponential transformation to the coefficients, and this transformation provides a more intuitive explanation of the relative change in survival time for each unit change in the metabolic factors. For example, blood glucose has the largest weight in the graph, indicating that for each unit increase in blood glucose, survival time decreases to 95.61% of its original value.

#### Randomised survival forest(RSF) model

As above, the RSF model based on machine learning algorithms was again used to assess the impact of each metabolic factor, with the relevant importance shown in Table 3, and a relative importance was established with weights for diabetes. It still shows that the metabolic factor of hyperglycemia has the greatest weight of importance.

## Discussion

This study investigated the influence of metabolic factors on the survival outcomes of MASLD patients. Analysis of the cohort comprising 3,086 MASLD patients yielded two significant findings. Firstly, although diagnostic criteria allow MASLD diagnosis based on a single metabolic factor, prognostic outcomes differ among patients according to the quantity and combinations of metabolic profile types. Both single and mixed exposure models showed consistent results, with hyperglycemia emerging as the strongest risk factor and hypertension as the secondary influential factor. The remaining three metabolic profiles were deemed comparatively weaker in their impact. A scoring formula was developed using a machine learning algorithm to facilitate a more scientific stratification of metabolic risk within this patient population.

MASLD involves dysregulated metabolic factors affecting the liver through multiple pathways. These interactions lead to diverse clinical manifestations and differing disease progression rates [15]. Extensive research has demonstrated a strong association between metabolism-related factors, such as type 2 diabetes mellitus, and the development of hepatic steatosis or fibrosis [18, 19]. The concomitant presence or sequential occurrence of multiple metabolic factors is associated with an increased severity of MASLD in patients. Mechanistically, multiple metabolic factors create greater lipid metabolism challenges compared to a single factor. This exacerbation results in the reprogramming of lipid metabolism [20], subsequently leading to increased lipid accumulation in the liver. The accumulated lipids induce oxidative stress and inflammation, impair hepatic fatty acid oxidation, and thereby facilitate the progression from MASLD to metabolic-associated steatohepatitis (MASH) [21, 22]. Research indicates that each additional metabolic profile elevates the risk of cirrhosis and hepatocellular carcinoma (HCC) in patients with MASLD [23], thereby contributing to decreased survival rates among this population. However, there is a notable paucity of studies examining patients with various combinations of metabolic profiles, and definitive investigations to elucidate the role of individual metabolic profiles within the metabolically dysfunctional cohort remain absent.

This study originated from a key clinical observation: MASLD patients show differing prognoses depending on specific combinations and counts of metabolic profile types. Notably, in the single-exposure model, hyperglycemia or hypertension showed significant correlations with reduced survival, whereas other metabolic factors lacked statistical significance. Meanwhile, following stratification based on varying numbers of metabolic profiles, an elevated mortality risk was observed across all strata for individuals presenting with either hyperglycemia, hypertension, or both. Consequently, there is substantial justification to posit that each metabolic factor, despite serving as an independent diagnostic criterion, does not contribute uniformly to metabolic dysfunction within this population. To achieve this objective, we employed an elastic network regression analysis of machine learning algorithms to develop a scoring formula that quantifies the joint contribution of each metabolic factor. Subsequently, we validated the weights of these factors from multiple perspectives utilizing both the random forest model and the accelerated time-to-failure model. Our findings indicated that hyperglycemia emerged as the most significant factor, followed by hypertension, while the remaining metabolic factors were comparatively less influential.

In addition to this, our subgroup analysis revealed a significant interaction between hyperglycemia and age. It was also found that younger patients (20–50 years old) had a higher risk than older age groups (HR = 2.08 vs. HR = 1.47). Although hypertension showed insignificant age interaction, young patients with hypertension had higher MASLD progression risk (HR = 1.97 vs. HR = 1.56). This age disparity may reflect accelerated hepatic fibrosis from prolonged metabolic stress exposure. Besides, female patients with hyperglycemia had higher MASLD progression risk (Female HR = 3.04 vs. 1.92 in males) and female hypertensive patients as the same (Female HR = 3.51 vs. HR = 1.99 in men), which may be related to the fact that, unlike the cardiovascular protection conferred by women in the general population, women with MASLD may lose this advantage due to a high metabolic load [24]. Another important interaction was seen between poverty level and hypertension and hyperglycemia, suggesting that socioeconomic status significantly modifies the effect of metabolic risk factors on the prognosis of MASLD. Specifically: the interaction between poverty index and hyperglycemia: patients in the lowest poverty index group (Pir < 1.3) had the highest risk of MASLD progression (HR = 3.51), which was significantly higher than that in the moderate (HR = 2.25) and high poverty index groups (HR = 2.08). The poverty-hypertension interaction showed similar significance, potentially stemming from: (1) suboptimal blood pressure/glucose control, (2) limited access to healthy diets, and (3) chronic stress aggravating metabolic dysfunction in low-resource environments. This suggests socioeconomic deprivation may intensify hyperglycemia’s hepatotoxic effects through constrained healthcare access and behavioral options. These findings support the development of individualized monitoring strategies and multidisciplinary intervention programs for high-risk subgroups (women, young patients, and low-income populations). Future research on molecular mechanisms needs to be combined with public health measures (e.g., community health screening) to reduce health disparities and optimize precision management of MASLD.

Some studies have also found that hyperglycemia is often associated with a state of insulin resistance and that insulin resistance is an important driver of fatty liver development [2, 25]. In an insulin-resistant state, the liver synthesizes more fat while inhibiting fat export, promoting fat accumulation in the liver [26]. At the same time, hyperglycemia increases oxidative stress and end products of glycosylation (AGEs) accumulation in the body by producing reactive oxygen species (ROS), which can bind to receptors on the cell surface (RAGE) to stimulate an inflammatory response [27, 28]. These factors can directly damage hepatocytes, exacerbate hepatic steatosis, and even progress to MASH, which may ultimately lead to cirrhosis or even hepatocellular carcinoma. Hypertension exerts dual effects: (1) elevated intravascular pressure mechanically damages hepatic vasculature, and (2) impaired endothelial dysfunction reduces hepatic perfusion. This combination exacerbates ischemic hepatocyte damage [29]. Abdominal obesity, on the other hand, is mainly characterized by an increase in abdominal fat, which is more likely to release fatty acids into the liver than other types of fat, thus increasing fat deposition in the liver [30]. However, the process of fat deposition is relatively slow and may require other synergistic factors (e.g., insulin resistance) to accelerate. Although abdominal obesity and hyperlipidaemia contribute to fatty liver development, their effects may be relatively weak due to the indirect nature of their mechanisms of action, their slower rate, and the need for synergistic effects of other metabolic mechanisms. Therefore, managing hyperglycemia and hypertension is particularly critical in the prevention and treatment of fatty liver, and the development of a composite score is suggestive of risk stratification for management and early intervention in patients with fatty liver.

In this study, hyperglycemia was found to be a central driver of survival risk in patients with MASLD, which exacerbates the process of hepatic lipid deposition and fibrosis through multiple mechanisms, including insulin resistance, oxidative stress, and dysregulation of the gut-liver axis. In clinical practice, it is imperative to prioritize the use of hepatoprotective hypoglycemic agents, such as GLP-1 agonists and SGLT2 inhibitors [31–33], in conjunction with adherence to a Mediterranean diet and engagement in low-intensity interval training to enhance metabolic outcomes [34, 35]. However, in patients with comorbid hypertension, another recent review of the literature suggests that antihypertensive medications have not yet had a beneficial effect on the progression of MASLD, and the specific effect awaits follow-up [36]. In addition, the metabolic risk scoring model developed in this study can be used to accurately stratify patients by integrating parameters: high-risk patients need to be initiated with early assessment of hepatic fibrosis (e.g., FibroScan) and multidisciplinary interventions (endocrinology, hepatology), with dynamic adjustment of glycemic control targets and monitoring of treatment efficacy. This model provides a feasible way to form a scientific tool for individualized treatment, and as a preliminary tool for individualized assessment of treatment, the model can be further optimized by combining multi-omics data and precise intervention strategies in the future.

This study possesses several strengths. Firstly, we employed a multifaceted methodological approach to investigate the effects of different metabolic factors on survival risk in MALSD patients, illustrating that survival within this population is contingent upon both the quantity and type of metabolic traits. More importantly, we utilized diverse machine learning algorithms to construct and validate a model that comprehensively evaluates the significance of each metabolic factor, and we developed formulas to calculate metabolic risk scores.

This study acknowledges several limitations that should be considered when interpreting the findings. While we examined the influence of select metabolic profiles on survival in patients with MASLD, it is possible that other metabolic factors not included in this assessment may also impact survival outcomes. Future research would benefit from incorporating a broader range of metabolic factors to enhance the predictive accuracy and validity of survival models in this population. Additionally, the retrospective nature of the study introduces potential biases, including selection bias and information bias, which are common in such study designs. The reliance on self-reported variables from NHANES III may further introduce inaccuracies, as self-reported data can be subject to recall bias and social desirability bias. These limitations may affect the precision of our estimates and the generalizability of our findings. Specifically regarding generalizability, while the NHANES cohort provides population-level insights, the clinical applicability of our risk model requires rigorous external validation in distinct clinical settings (e.g., hospital-based cohorts) and geographic populations to assess spectrum bias and transportability. Furthermore, practical implementation barriers must be acknowledged, including interoperability challenges with electronic health record systems and temporal availability of specialized biomarkers in routine clinical practice. Moreover, the absence of detailed survival information in the database constrained our analysis, particularly limiting our ability to conduct a comprehensive assessment of liver-related mortality. The unavailability of such specific mortality data in the attributable Cox analysis represents another significant limitation, potentially leading to residual confounding. Given these constraints, the results should be interpreted with caution. While efforts were made to adjust for potential confounders, unmeasured confounding variables may still influence the observed associations. Importantly, the clinical implications should be contextualized within these methodological constraints. Future validation studies should adhere to TRIPOD guidelines to evaluate both discrimination and calibration across diverse populations. Concurrent implementation science research is needed to address system-level barriers, including development of point-of-care calculation tools and pilot testing through quality improvement initiatives. Future research with prospective study designs, objective measurement of metabolic factors, and complete mortality data, including liver-related mortality, is necessary to further validate and expand upon our findings.

## Conclusions

In conclusion, this study robustly demonstrates that metabolic factors, particularly elevated blood glucose levels and blood pressure, are closely linked to increased mortality rates among patients with MASLD. Utilizing machine learning algorithms, our research has systematically quantified the differential impacts of various metabolic dysfunctions on patient survival, revealing the complex interplay and potential underlying metabolic mechanisms at play. Importantly, the development of a metabolic scoring system based on these findings represents a transformative step forward. This scoring system not only offers a practical tool for assessing patient health status but also enhances the ability to predict survival outcomes in clinical settings. The early identification and stratification of high-risk patients through this scoring mechanism can facilitate timely and targeted interventions, potentially improving survival rates and paving the way for personalized therapeutic strategies in the management of MASLD. This study sets a foundation for future research to explore and refine these interventions, emphasizing its significant clinical relevance and prospective impact on healthcare.This proactive approach in patient management could transform standard care practices, leading to better health outcomes and more efficient use of medical resources. Future research could focus on validating and refining this scoring system to ensure its efficacy across diverse populations, with the aim of integrating it into routine clinical workflows.

## Electronic supplementary material

Below is the link to the electronic supplementary material.

Supplementary Material 1: Figure S1: Kaplan-Meier curves by hypertension (HBP) or hyperglycemia (GLU) status in MASLD patients. Red/blue curves represent patients with/without HBP or GLU, respectively, showing survival probabilities over time. P value evaluates between-group differences, highlighting the impact of these metabolic factors on survival outcomes.

Supplementary Material 2: Figure S2: Survival curves stratified by metabolic factors (1-5). illustrating survival probabilities over time across different combinations. Abbreviations in the figure indicate relevant metabolic factors: GLU, hyperglycemia; HBP, hypertension; BMI, abdominal obesity; TG, hypertriglyceridemia; HDL, low HDL-cholesterolemia.

Supplementary Material 3: Figure S3: Forest plots of subgroup analyses. Assessing associations between abdominal obesity, elevated TG, and reduced HDL-C with all-cause mortality in MASLD patients. Boxes represent HR point estimates (size reflects subgroup weight); horizontal lines show 95% CIs (statistical significance if CI excludes 1). P values and P for interaction denote association strength and subgroup effect differences, respectively. Elevated HRs indicate higher mortality risk.

Supplementary Material 4: Figure S4: Elastic Net Regression Model Partial Likelihood Deviance Plot. This plot shows the relationship between log(lambda) and partial likelihood deviance. The dashed vertical lines mark the lambda values for minimum deviance (12.33) and 1SE deviance (12.39). The minimum deviance point?Pink Vertical Line? represents the lambda value that best balances model complexity and goodness of fit. The 1SE deviance point offers one standard error of the minimum deviance.

Supplementary Material 5: Figure S5: Kaplan-Meier survival curves grouped by metabolic-related survival risk score quartiles. Patients were divided into four groups based on their metabolic scores, and survival probabilities over time are shown for each group. The curves illustrate how survival probabilities differ across metabolic score quartiles, highlighting the relationship between metabolic score and survival outcomes.

Supplementary Material 6: Table S1: Hazard Ratios for cardiovascular and cancer attribution grouped according to the number of metabolic factors.

Supplementary Material 7: Table S2: Hazard Ratios for quartiles of metabolic-related survival risk score.

## Data Availability

No datasets were generated or analysed during the current study.
