# Supplementary figures and images for: Decoding survival in MASLD: the dominant role of metabolic factors
Source: Diabetol Metab Syndr. 2025 Jun 18;17:226. doi: 10.1186/s13098-025-01802-9 (PMC12175318; doi:10.1186/s13098-025-01802-9)

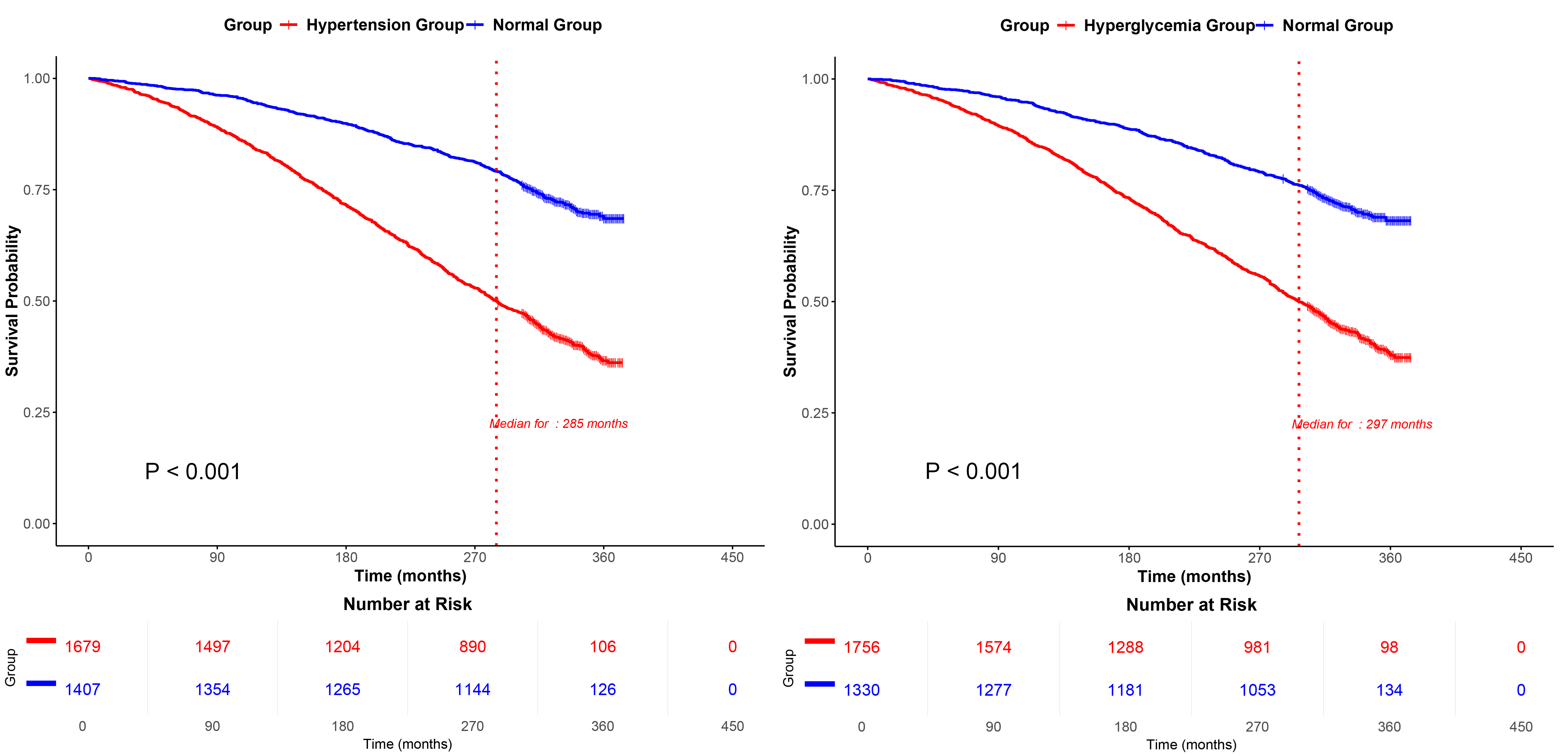

Supplement: Supplementary file 1 — Supplementary Material 1: Figure S1: Kaplan-Meier curves by hypertension (HBP) or hyperglycemia (GLU) status in MASLD patients. Red/blue curves represent patients with/without HBP or GLU, respectively, showing survival probabilities over time. P value evaluates between-group differences, highlighting the impact of these metabolic factors on survival outcomes. [file 13098_2025_1802_MOESM1_ESM.tif]

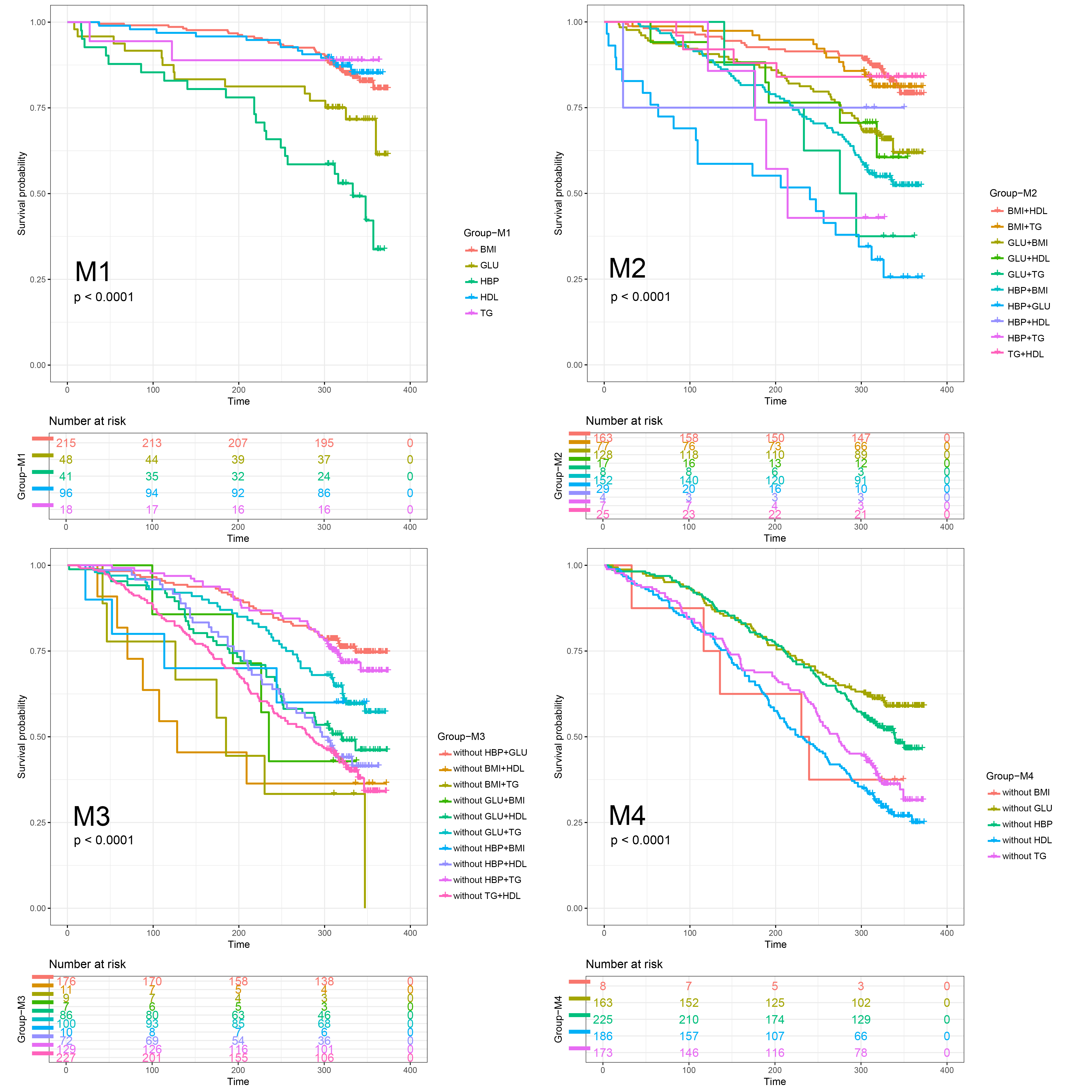

Supplement: Supplementary file 2 — Supplementary Material 2: Figure S2: Survival curves stratified by metabolic factors (1-5). illustrating survival probabilities over time across different combinations. Abbreviations in the figure indicate relevant metabolic factors: GLU, hyperglycemia; HBP, hypertension; BMI, abdominal obesity; TG, hypertriglyceridemia; HDL, low HDL-cholesterolemia. [file 13098_2025_1802_MOESM2_ESM.tif]

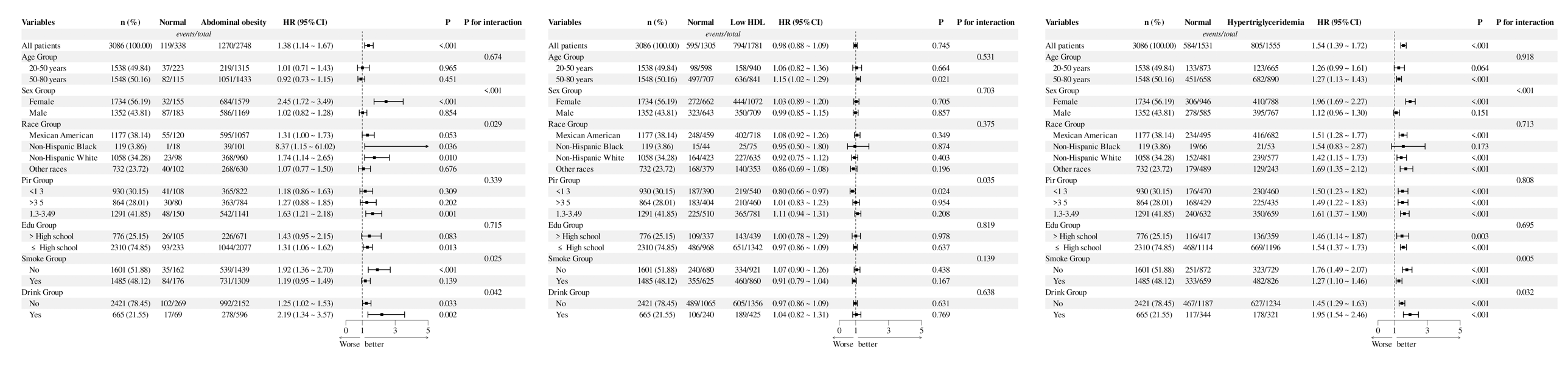

Supplement: Supplementary file 3 — Supplementary Material 3: Figure S3: Forest plots of subgroup analyses. Assessing associations between abdominal obesity, elevated TG, and reduced HDL-C with all-cause mortality in MASLD patients. Boxes represent HR point estimates (size reflects subgroup weight); horizontal lines show 95% CIs (statistical significance if CI excludes 1). P values and P for interaction denote association strength and subgroup effect differences, respectively. Elevated HRs indicate higher mortality risk. [file 13098_2025_1802_MOESM3_ESM.tif]

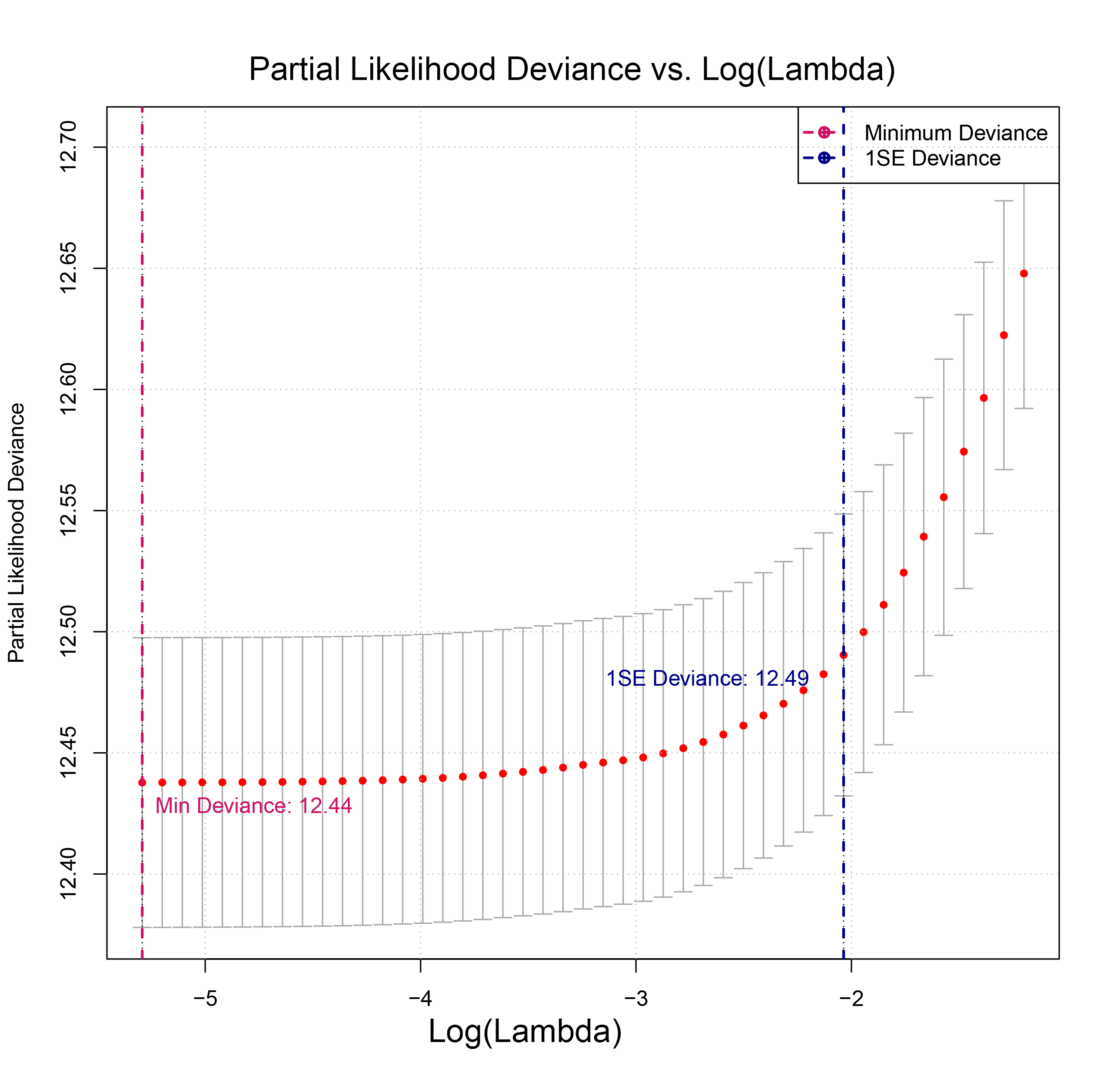

Supplement: Supplementary file 4 — Supplementary Material 4: Figure S4: Elastic Net Regression Model Partial Likelihood Deviance Plot. This plot shows the relationship between log(lambda) and partial likelihood deviance. The dashed vertical lines mark the lambda values for minimum deviance (12.33) and 1SE deviance (12.39). The minimum deviance point?Pink Vertical Line? represents the lambda value that best balances model complexity and goodness of fit. The 1SE deviance point offers one standard error of the minimum deviance. [file 13098_2025_1802_MOESM4_ESM.tif]

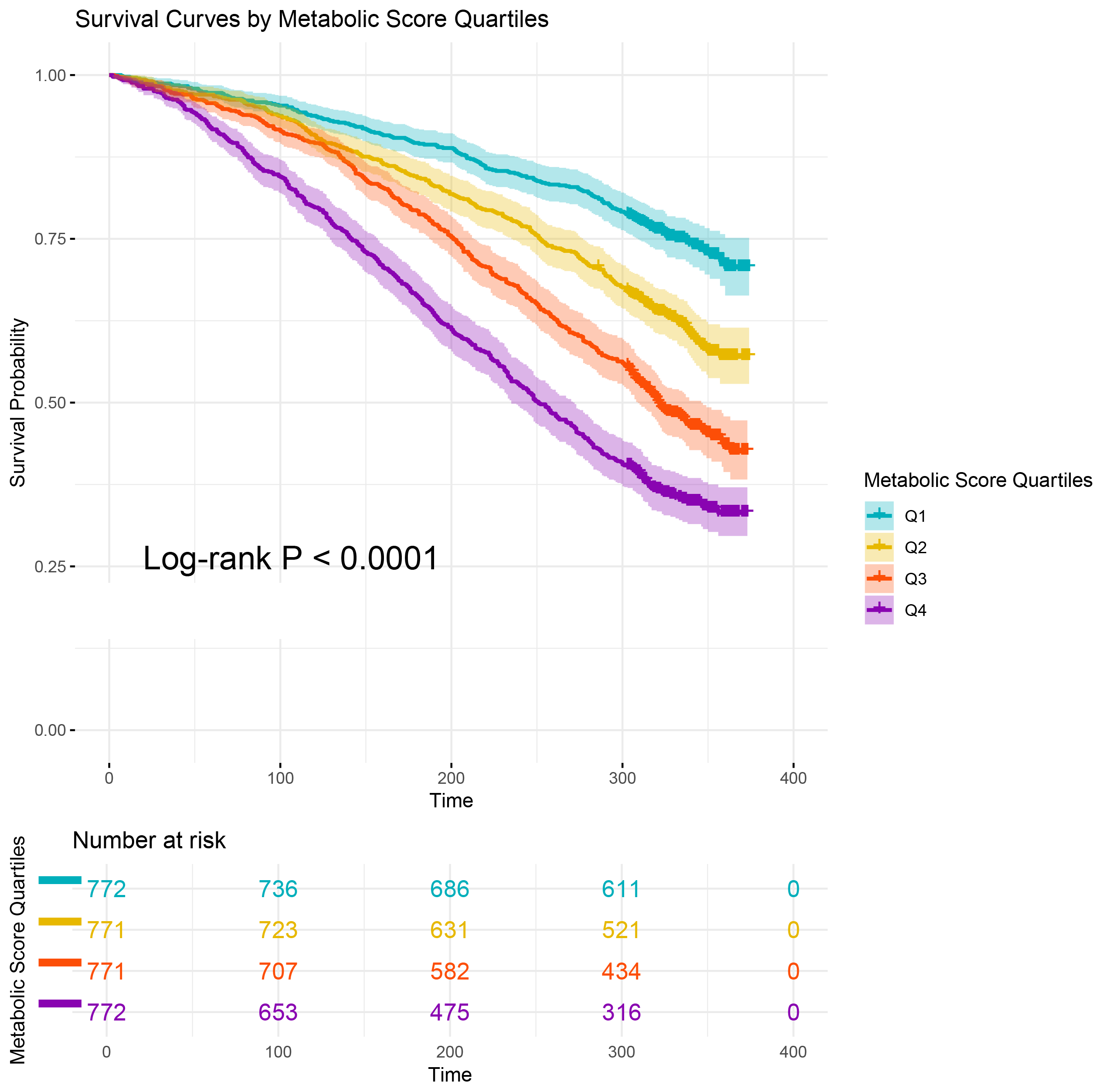

Supplement: Supplementary file 5 — Supplementary Material 5: Figure S5: Kaplan-Meier survival curves grouped by metabolic-related survival risk score quartiles. Patients were divided into four groups based on their metabolic scores, and survival probabilities over time are shown for each group. The curves illustrate how survival probabilities differ across metabolic score quartiles, highlighting the relationship between metabolic score and survival outcomes. [file 13098_2025_1802_MOESM5_ESM.tif]
