# Supplementary material for: Decoding survival in MASLD: the dominant role of metabolic factors
Source: Diabetol Metab Syndr. 2025 Jun 18;17:226. doi: 10.1186/s13098-025-01802-9 (PMC12175318; doi:10.1186/s13098-025-01802-9)
Supplement: Supplementary file 6 — Supplementary Material 6: Table S1: Hazard Ratios for cardiovascular and cancer attribution grouped according to the number of metabolic factors. [file 13098_2025_1802_MOESM6_ESM.docx]

STable 1: Hazard Ratios for cardiovascular and cancer attribution were grouped according to the number of metabolic factors

|  | **Model 1** | | | **Model 2** | | | **Model 3** | | |
| --- | --- | --- | --- | --- | --- | --- | --- | --- | --- |
| cardiovascular | **HR**^1^ | **95% CI**^1^ | **p-value** | **HR**^1^ | **95% CI**^1^ | **p-value** | **HR**^1^ | **95% CI**^1^ | **p-value** |
| M1 | Reference | | | Reference | | | Reference | | |
| M2 | 2.03 | 1.16, 3.55 | 0.014 | 1.07 | 0.59, 1.93 | 0.821 | 1.18 | 0.65, 2.16 | 0.581 |
| M3 | 1.84 | 1.09, 3.10 | 0.023 | 1.11 | 0.64, 1.91 | 0.718 | 1.26 | 0.71, 2.22 | 0.428 |
| M4 | 2.16 | 1.29, 3.62 | 0.004 | 1.27 | 0.73, 2.20 | 0.401 | 1.42 | 0.81, 2.52 | 0.223 |
| M5 | 2.07 | 1.23, 3.46 | 0.006 | 1.14 | 0.66, 1.97 | 0.633 | 1.30 | 0.74, 2.28 | 0.367 |
| *P* for trend |  |  | 0.006 |  |  | 0.455 |  |  | 0.348 |
| Malignant neoplasms | **Model 1** | | | **Model 2** | | | **Model 3** | | |
| M1 | Reference | | | Reference | | | Reference | | |
| M2 | 1.27 | 0.78, 2.07 | 0.340 | 1.14 | 0.69, 1.90 | 0.608 | 1.12 | 0.67, 1.87 | 0.669 |
| M3 | 1.20 | 0.78, 1.83 | 0.406 | 0.79 | 0.51, 1.24 | 0.305 | 0.77 | 0.49, 1.20 | 0.248 |
| M4 | 1.34 | 0.88, 2.04 | 0.166 | 0.95 | 0.61, 1.47 | 0.804 | 0.92 | 0.59, 1.43 | 0.700 |
| M5 | 1.46 | 0.92, 2.33 | 0.108 | 1.04 | 0.64, 1.72 | 0.866 | 1.01 | 0.60, 1.68 | 0.977 |
| *P* for trend |  |  | 0.110 |  |  | 0.085 |  |  | 0.085 |
| ^1^HR = Hazard Ratio, CI = Confidence Interval | | | | | | | | | |

Adjust:

Model 1: Unadjusted

Model 2: Adjusted for age, sex, race/ethnicity, poverty degree, education level, smoking status;

Model 3: Adjusted for age, sex, race/ethnicity, poverty degree, education level, smoking status, and alanine aminotransferase (ALT);
