# Supplementary material for: Decoding survival in MASLD: the dominant role of metabolic factors
Source: Diabetol Metab Syndr. 2025 Jun 18;17:226. doi: 10.1186/s13098-025-01802-9 (PMC12175318; doi:10.1186/s13098-025-01802-9)
Supplement: Supplementary file 7 — Supplementary Material 7: Table S2: Hazard Ratios for quartiles of metabolic-related survival risk score. [file 13098_2025_1802_MOESM7_ESM.docx]

STable 2: Hazard Ratios for quartiles of metabolic-related survival risk score

|  | **Model 1** | | | **Model 2** | | | **Model 3** | | |
| --- | --- | --- | --- | --- | --- | --- | --- | --- | --- |
| Metabolism-related survival risk score | **HR**^1^ | **95% CI**^1^ | **p-value** | **HR**^1^ | **95% CI**^1^ | **p-value** | **HR**^1^ | **95% CI**^1^ | **p-value** |
| **Q1** | Reference | | | Reference | | | Reference | | |
| **Q2** | 1.65 | 1.38, 1.98 | <0.001 | 1.10 | 0.91, 1.32 | 0.3 | 1.09 | 0.91, 1.31 | 0.3 |
| **Q3** | 2.47 | 2.08, 2.93 | <0.001 | 1.33 | 1.11, 1.58 | 0.002 | 1.32 | 1.11, 1.58 | 0.002 |
| **Q4** | 3.65 | 3.09, 4.31 | <0.001 | 1.90 | 1.60, 2.26 | <0.001 | 1.90 | 1.60, 2.25 | <0.001 |
| *P* for trend |  |  | <0.001 |  |  | <0.001 |  |  | <0.001 |
| ^1^HR = Hazard Ratio, CI = Confidence Interval | | | | | | | | | |

Adjust:

Model 1: Unadjusted

Model 2: Adjusted for age, sex, race/ethnicity, poverty degree, education level, smoking status;

Model 3: Adjusted for age, sex, race/ethnicity, poverty degree, education level, smoking status, and alanine aminotransferase (ALT);
